# BMP4, a new prognostic factor for glioma

**DOI:** 10.1186/1477-7819-11-264

**Published:** 2013-10-08

**Authors:** Qiang Wu, Jiarui Yao

**Affiliations:** 1 Department of Neurology, Xinxiang Central Hospital, 56 Jin Hui Da Street, Henan 453000, China; 2 Department of Neurology, Chinese PLA General Hospital, 28 Fuxin Road, Beijing 100853, China

**Keywords:** Glioma, RT-PCR, Immunohistochemistry, BMP4, Survival rate

## Abstract

**Background:**

The expression status of bone morphogenetic protein 4 (BMP4) in gliomas is still unclear by now. We try to investigate the relationship between BMP4 expression and the biological behavior of gliomas in order to lay a foundation for the management of these tumors.

**Methods:**

A total of 630 patients with glioma were enrolled in the study from January 2002 to January 2008. The expression status of BMP4 in gliomas was evaluated by RT-PCR and immunohistochemistry. The relationships between BMP4 expression and clinicopathological parameters and between BMP4 expression and prognosis were also studied.

**Results:**

The expression of BMP4 in tumor tissues was significantly lower than that in the paracancer tissues at both mRNA and protein levels (*P* = 0.01 and 0.001, respectively). Univariate analysis showed that BMP4 expression was closely related to extent of resection, Ki-67 expression, and the WHO grade (*P* = 0.001, 0.001, and 0.001, respectively), but it was not related to age, sex, or the Karnofsky Performance Status (KPS) score (*P* = 0.099, 0.472, and 0.201, respectively). Finally, Ki-67 expression and the WHO grade were found to be related to BMP4 expression using logistic regression (*P* = 0.001 and 0.001, respectively). Interestingly, we found that the expression of BMP4 was significantly related to distant glioma metastasis. Cox regression analysis identified the KPS score, extent of resection, Ki-67 expression, WHO grade, and BMP4 expression as independent prognostic factors (*P* = 0.044, 0.010, 0.002, 0.001, and 0.001, respectively).

**Conclusions:**

BMP4 is differentially expressed in glioma patients and is closely related to the biological behavior of gliomas. BMP4 expression was found to be a strong predictor of distant metastasis and postoperative prognosis.

## Background

A glioma is a type of tumor that starts in the brain or spine. Gliomas make up approximately 30% of all brain and central nervous system tumors and 80% of all malignant brain tumors [1]. They are fast growing and have a high recurrence rate [2]. The five-year survival rate for patients with glioma is low even with surgery, radiotherapy, chemotherapy, and other forms of treatment [3]. Because of the infiltrative growth of gliomas, it is difficult to resect the whole tumor without causing serious damage to brain function [4]. The use of molecular biology methods to explore the pathogenic basis of gliomas may yield further insight into the treatment of this disease.

Bone morphogenetic proteins (BMPs) are multifunctional growth factors that belong to the transforming growth factor beta (TGF-β) superfamily [5]. Members of the TGF-β superfamily are synthesized as precursor macromolecules, and the mature protein is released from the propeptide by proteolytic cleavage [6]. BMPs have been grouped into 15 categories, which are divided into three subsets according to amino acid sequence: BMP2 and BMP4, BMP5 and BMP8, and BMP3 and GDF10. BMP4 expression is closely related to the grade of malignancy in gastric carcinoma [7].

A characteristic of brain gliomas is the presence of stem-like cells in the tumor tissue that can develop into any kind of cell when stimulated by the correct ‘signal’ [8]. A recent study found that BMP4 can cause these neural stem-cell-like clusters to lose their brood cell characteristics, thereby terminating their ability to differentiate [9]. All mice infected with untreated glioma cells died after three to four months, but nearly all mice infected with BMP4-treated cells survived.

At present, the expression status of BMP4 in gliomas and the mechanism of BMP4 action are unclear, and studies investigating the function and mechanism of this molecule in the biological behavior of gliomas are still rare. We studied the relationship between BMP4 expression and the biological behavior of gliomas in order to provide a theoretical basis for the treatment of glioma.

## Methods

### Clinical specimens and experimental materials

Paraffin-embedded specimens of brain gliomas from 630 patients were collected at Liaoning Provincial Tumor Hospital and Xinxiang Central Hospital from January 2002 to January 2008. These cases were used for testing of protein levels by immunohistochemistry and for analysis of patient prognosis. The average age of enrolled patients was 46.33 ± 8.49 years (range from 34 to 77 years). Gliomas can be divided into low-grade (World Health Organization (WHO) grade II) and high-grade gliomas (WHO grade III-IV) depending on their rate of growth [10]. Based on histopathological examination, patients were divided into two groups: 247 with low-grade gliomas and 383 with high-grade gliomas. All patients were assessed by the Karnofsky Performance Status (KPS) scale: (1) minor disability (80 to 100 points); (2) moderate disability (60 to 70 points); and (3) severe disability (10 to 50 points) [11].

The inclusion criteria were as follows: (a) resected specimens underwent pathological examination, (b) enrolled cases were successfully followed up, and (c) a complete medical record was available. The study protocol was approved by the Ethics Committee of Xinxiang Central Hospital and written informed consent was obtained from patients in the study.

### RT-PCR

Total RNA was isolated using TRIzol Reagent (Life Technologies, Carlsbad, CA, USA) according to the manufacturer’s instructions. cDNAs were synthesized using a RevertAid First Strand cDNA Synthesis Kit (Fermentas,Vilnius, Lithuania). Quantitative real-time PCR was performed using SYBR Green PCR Master Mix on an Applied Biosystems (Foster City, CA, USA) 7500 Fast Real-Time PCR System. Primers used in the real-time PCR were as follows: forward 5′-AGCAGCCAAACTATGGGCTA-3′ and reverse 5′-TGGTTGAGTTGAGGTGGTCA-3′. The reaction conditions were 95°C for 5 min followed by 40 cycles of 94°C for 15 s, and 55.5 or 55.2°C for 20 s. Melting temperature curve analyses were performed after PCR.

### Western blot

Protein concentrations were determined by bicinchoninic acid (BCA) assay (Santa Cruz Biotechnology, Santa Cruz, CA, USA). Equal amounts of total protein were loaded and separated by SDS-PAGE and transferred onto polyvinylidene fluoride(PVDF) membranes. Membranes were blocked using 5% fat-free milk in phosphate-buffered saline (PBS) at 37°C for 2 h, followed by two washes with phosphate-buffered saline/Tween (PBST). Blots were then incubated with primary antibodies to BMP4 (1:500; Cell Signaling Technology, Beverly, MA, USA) or glyceraldehyde-3-phosphate dehydrogenase (GAPDH) (1:800; Santa Cruz Biotechnology) at 4°C overnight and washed four times with PBST before adding secondary antibody (horseradish peroxidase (HRP)-conjugated anti-rabbit or anti-mouse or anti-goat immunoglobulin (Ig)G). Blots were incubated with secondary antibody for 1 h at room temperature and then washed four times with PBST. Protein bands were visualized by chemiluminescence and the target protein quantity was determined by normalizing the densities of corresponding bands to those of the loading control bands (GAPDH).

### Immunohistochemistry

Tumor tissue microarray blocks were freshly cut into 4-μm thick sections. Sections were fixed on slides and dried for 12 to 24 h at 37°C. Sections were subsequently deparaffinized in xylene and rehydrated through a graded series of ethanol to distilled water. After antigen retrieval, sections were incubated for 60 min with primary antibody to Ki-67 (sc-15402; Santa Cruz Biotechnology) or BMP4 (1:500; Cell Signaling Technology). Following washing with PBS, sections were incubated for 30 min with the biotinylated secondary antibody (multi-link swine anti-goat/mouse/rabbit immunoglobulin; Dako, Glostrup, Denmark). After washing, Avidin/Biotin Complex (1:1000; Vector Laboratories, Burlingame, CA, USA) was applied to the sections for 30 to 60 min at room temperature. The sections were washed and the immunoreactive products were visualized by oxidation of 3,3′-diaminobenzidine (DAB) by HRP in the presence of H_2_O_2_. Sections were then counter stained in Gill’s hematoxylin and dehydrated in ascending grades of methanol before clearing in xylene and mounting under a coverslip.

For negative controls, the primary antibody was replaced by 0.01 mol/L PBS, and for positive controls, normal breast tissue sections were used. Cells positive for BMP4 were defined as those with brown granules in the cytoplasm. Two hundred cells from two representative fields of each section were counted by two independent observers to determine the immunostaining intensity. Staining intensity was recorded on a 4-point scale: 0, no staining; 1, light brown (weak immunostaining); 2, brown (moderate immunostaining); 3, dark brown (strong immunostaining). Also, the extent of staining was assessed on a 3-point scale: 0, <9% positive cells; 1, 10 to 50% positive cells; 2, >50% positive cells. Scores from the two scales were combined and each section was classified as low/no BMP4 expression (0 to2) or high BMP4 expression [3-6]. Ki-67 expression was classified semi-quantitatively according to the following criteria: samples were considered positive for Ki-67 if more than 10% of neoplastic cells discretely expressed Ki-67 in their nucleus.

### Statistical analysis

All data were analyzed with SPSS Statistics software (version 13.0; SPSS, Inc., Chicago, IL, USA). The relationships between BMP4 and other parameters were studied using the chi-square test, Fisher’s exact test, or independent *t* tests. Disease-specific survival was analyzed using the Kaplan-Meier method. The log-rank test was used to analyze differences in survival. Multivariate analysis was performed using the Cox proportional hazards model with forward stepwise selection. A *P* value of less than 0.05 was considered statistically significant.

## Results

### BMP4 expression in human glioma tissues at mRNA and protein levels

**Figure 1:**
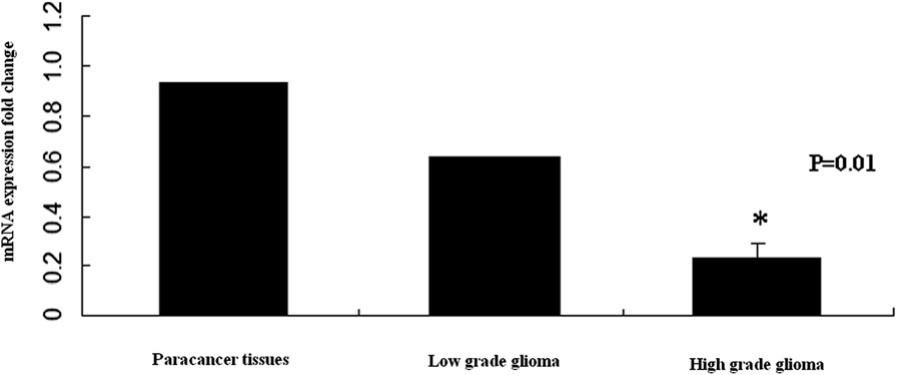
**RT-PCR analysis showed that BMP4 mRNA was upregulated in paracancer tissues compared with low-grade or high-grade glioma tissues (*P *= 0.01).**

**Figure 2:**
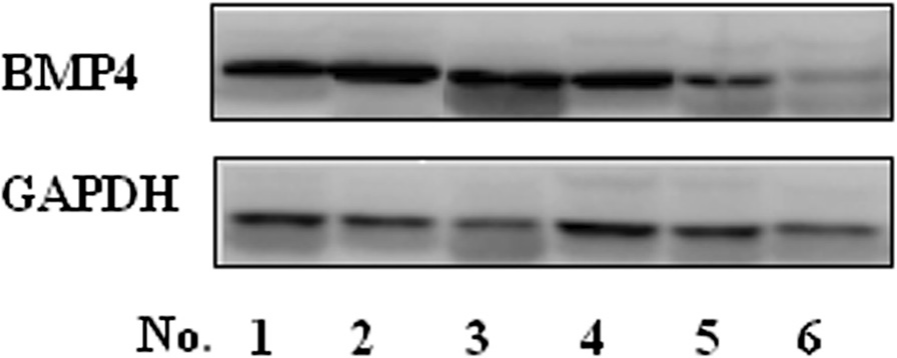
**Western blot analysis showed that BMP4 protein was upregulated in paracancer tissues (No. 1, 2) compared with glioma tissues and in low-grade glioma tissues (No. 3, 4)compared with high-grade glioma tissues (No. 5, 6) (*P *= 0.001).**

RT-PCR analysis of high-grade (WHO III-IV) and low-grade (WHO I-II) tumor tissues showed that BMP4 mRNA expression was significantly lower in tumor tissues than in paracancer tissues (Figure 1). Furthermore, BMP4 mRNA was upregulated in low-grade glioma tissues when compared with high-grade glioma tissues (*P* = 0.01) (Figure 1). Furthermore, in western blot analysis, the BMP4 protein was upregulated in low-grade glioma tissues when compared with high-grade glioma tissues (*P* = 0.001). Moreover, BMP4 protein expression in tumor tissues was significantly lower than that inparacancer tissues (*P* = 0.001) (Figure 2).

### Expression of BMP4 ingliomas and the relationship between BMP4 expression and clinicopathological characteristics

**Figure 3:**
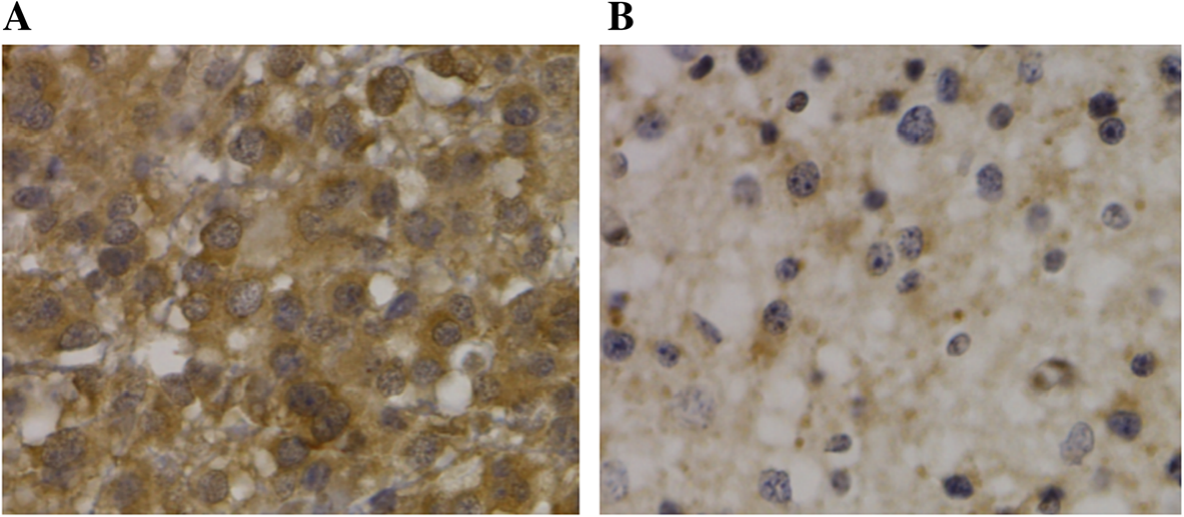
**Expression of BMP4 in glioma tissues (×400). (A)** Low-grade glioma stained for BMP4; **(B)** high-grade gliomas stained for BMP4.

Immunohistochemical analyses showed that BMP4 localized in the cytoplasm of glioma cells (Figure 3). BMP4 expression was observed in 36.98% (233/630) of the glioma sections. Univariate analysis showed that BMP4 expression was closely related to the extent of resection, Ki-67 expression, and the WHO grade (*P* = 0.001, 0.001, and 0.001, respectively), but it was not related to patient age, gender, or KPS score (*P* = 0.099, 0.472, and 0.201, respectively) (Table 1). Finally, Ki-67 expression and the WHO grade were found to be related to BMP4 expression using logistic regression (*P* = 0.001 and 0.001, respectively) (Table 2).

**Table 1:** Relationship between BMP4 expression and clinic-pathological factors of 630 gliomas

**Variable**	**n**	**BMP4 (n(%))**		**X**^**2**^	***P *value**
		**+**	**-**		
Sex				0.517	0.472
Male	347	124(35.73)	223(64.27%)		
Female	283	109(38.52)	174(61.48)		
Age (years)				2.982	0.099
≤35	307	124(40.39)	183(59.61)		
>35	323	109(33.75)	214(66.25)		
KPS score				3.208	0.201
10-50	83	38(45.78)	45(54.22)		
60-70	135	49(36.30)	86(63.70)		
80-100	412	146(35.44)	266(64.56)		
Extent of resection				21.702	0.001
Partial	139	28(20.14)	111(79.86)		
Total	491	205(41.75)	286(58.25)		
Ki67				78.795	0.001
+	291	54(18.56)	237(81.44)		
_	339	179(52.80)	160(47.20)		
WHO grade				40.996	0.001
II	247	128(51.82)	119(48.18)		
III-IV	383	105(27.42)	278(72.58)		

+, high BMP4 expression; –, loss/low BMP4 expression. KPS, Karnofsky Performance Status; WHO, World Health Organization.

**Table 2:** Multivariate analysis of the factors related to BMP4 expression

**Characteristic**	**Hazard ratio**	**95% CI**	***P *value**
Sex	1.147	0.683-1.925	0.604
Age	0.998	0.624-1.597	0.998
KPS	1.140	0.843-1.541	0.396
Extent of resection	0.581	0.369-2.728	0.182
Ki67	1.605	1.241-2.076	0.001
WHO grade	3.901	1.705-8.928	0.001
Constant	1.704		

CI, confidence interval; KPS, Karnofsky Performance Status; WHO, World Health Organization.

### Prognostic analysis

**Figure 4:**
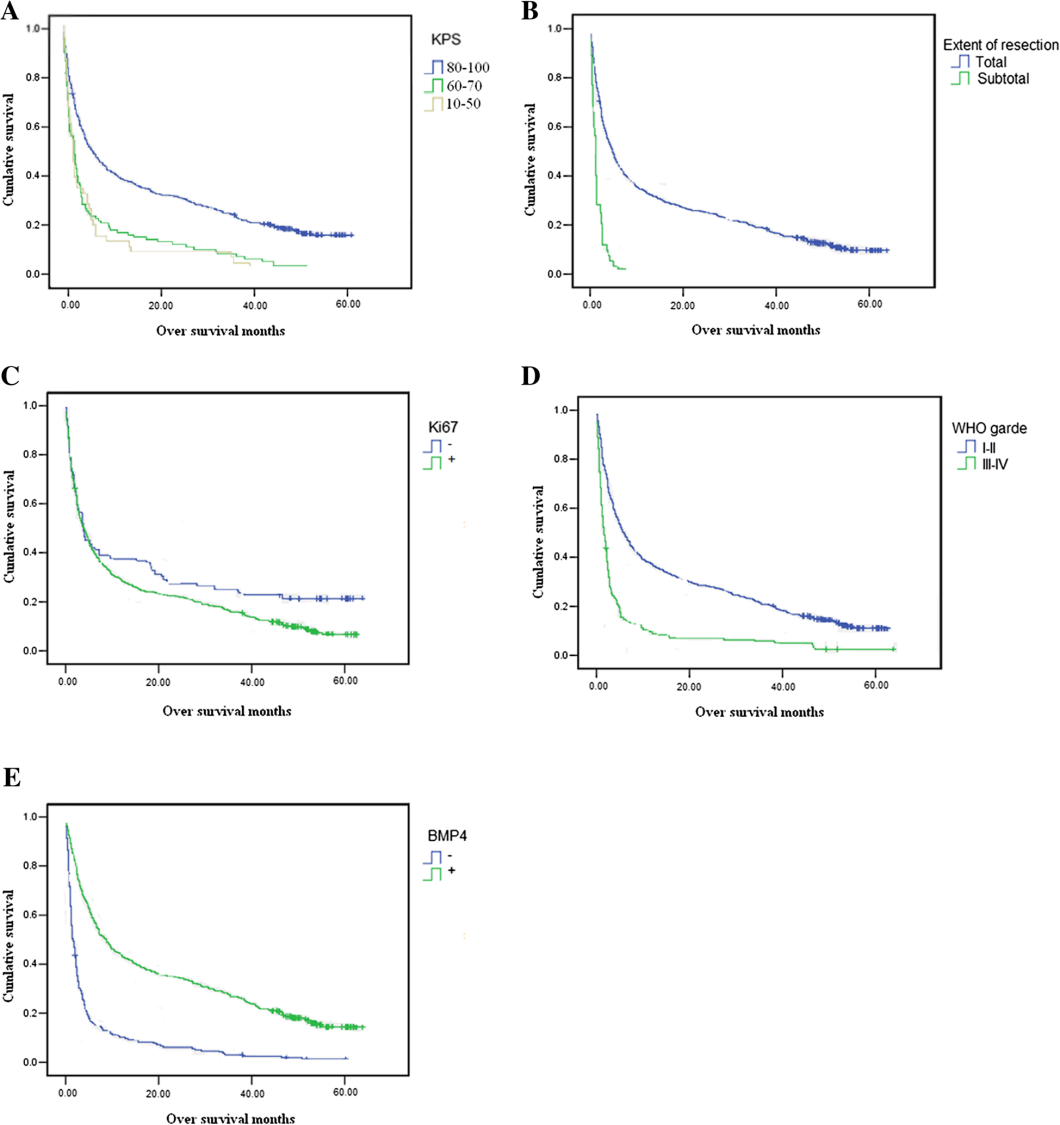
**Survival analysis of prognostic factors of glioma.** KPS score **(A)**, extent of resection **(B)**, Ki-67 expression **(C)**, WHO grade **(D)**, and BMP4 expression **(E)** as independent prognostic factors (P = 0.02, 0.001, 0.001, 0.001, and 0.001, respectively).

Survival analysis showed that patients with high BMP4 expression had significantly poorer postoperative disease-specific survival than those with low/no BMP4 expression (*P* = 0.001) (Figure 4). Cox regression analysis identified the KPS score, extent of resection, Ki-67 expression, WHO grade, and BMP4 expression as independent prognostic factors (*P* = 0.044, 0.010, 0.002, 0.001, and 0.001, respectively).

## Discussion

BMP4 plays an important role in the development of the nervous system [12]. It can induce neural stem cells to differentiate into either neurons or astrocytes and may affect neural stem cell differentiation through multiple mechanisms. Although the basic function of BMP4 is to induce bone formation, it is reported to play a role in the development and metastasis of malignant tumors [13]. BMP4 expression has been reported in osteosarcoma, bone fibrous dysplasia, gastric cancer, prostate cancer, and salivary gland tumors, and the expression of this protein may affect the biological behavior of tumors and the disease prognosis [14-16]. Gajera *et al.*[17] reported that the biological behavior of osteosarcoma cells with high BMP4 expression was obviously different from that of cells with low or no BMP4 expression [17,18]. In another study, BMP4 also showed the two sides of oncogenes and anti-oncogenes in breast cancer [19]. Kallioniemi *et al.*[20] reported that BMP4 could inhibit the growth of breast cancer cells but could also induce these cells to migrate, invade, and undergo epithelial to mesenchymal transformation both *in vivo* and *in vitro*[20]. Montesano *et al.*[21] reported that at concentrations of 1 to 10 ng/mL, BMP4 could inhibit lumen formation by epithelial cells *in vitro*, while at higher concentrations (20 to 100 ng/mL) it could disrupt cell-cell adhesion, resulting in disorganization of the epithelial cysts and the spreading of individual cells to the surrounding collagenous matrix [21].

In the nervous system, BMP4 plays a role in maintaining and regulating the health and regeneration of peripheral nerves. It acts as aneurotrophic factor that may be involved in the occurrence and development of tumors of the nervous system. A heterogeneity in BMP4 expression depending on disease type, from benign disease to malignant tumors [6,17-19], has been observed. BMP4 may also play different roles during different tumor stages, but its mechanism in both benign and malignant tumors of the nervous system [20,21] requires further study.

Glioma prognosis is often poor, even when patients undergo surgery combined with radiotherapy and chemotherapy. Research priorities over the next 10 years will be focused on both basic and translational approaches. New treatment methods may include new drugs that block cell proliferation-inducing signaling pathways to overcome resistance to chemotherapy. In some studies, BMP4 has been observed to promote the differentiation of glioma stem cells, inhibit their proliferation, and decrease tumorigenicity [6,9]. It may rely on signal transducer and activator of transcription(STAT) signaling. In this function, BMP4 is the most potent member of the BMP family. Piccirillo *et al.*[9] reported that BMP4 can inhibit growth of glioma cells [9].

A recent study found that BMP4 can cause neural stem-cell-like clusters to lose their brood cell characteristics, which interferes with their ability to divide. Cells treated with BMP4 did not produce tumors in infected mice, whereas mice infected with untreated cells died [6]. But in clinical cases, the effect of BMP4 expression on the prognosis for glioma is still unclear. We found that the expression of BMP4 in low-grade gliomas was significantly higher than that in high-grade gliomas. BMP4 expression was closely related to Ki-67 expression and WHO grade but was not correlated with age, sex, KPS score, or extent of resection. Interestingly, we found that high BMP4 expression can increase bone metastasis from brain glioma. We conclude that BMP4 is an independent prognostic factor for glioma.

## Conclusion

Differential expression of BMP4 is closely related to the biological behavior of brain gliomas. It is an independent prognostic factor for glioma that can provide a basis for clinical treatment of brain glioma.

## Competing interests

The authors declare that they have no competing interests.

## Authors’ contribution

QW and JY carried out the RT-PCR and immunohistochemistry studies, Wu done the survival analysis and drafted the manuscript. Both authors read and approved the final manuscript.
